# Ester-Modified Sodium Silicate Grout Material for Moraine Stabilization: Synthesis and Freeze-Thaw Resistance

**DOI:** 10.3390/ma17225512

**Published:** 2024-11-12

**Authors:** Chong Chen, Aixiang Wu, Shaoyong Wang, Wei Sun, Tong Gao, Longjian Bai

**Affiliations:** 1 School of Civil and Resource Engineering, University of Science and Technology Beijing, Beijing 100083, China; 2 Faculty of Land and Resources Engineering, Kunming University of Science and Technology, Kunming 650093, China

**Keywords:** grout materials, ester-modified sodium silicate, moraine, freeze-thaw resistance, consolidated body

## Abstract

To achieve effective consolidation of fine particles in moraine and enhance the freeze-thaw resistance of the consolidated body, this study developed a novel grouting material using sodium silicate, lipid-based curing agents, and acidic catalysts. The gelation time and rheological properties of this material were tested. The freeze-thaw resistance was studied through changes in uniaxial compressive strength (UCS) after freeze-thaw cycles, while the consolidation mechanism was analyzed using X-ray diffraction (XRD), Fourier-transform infrared spectroscopy (FTIR), and scanning electron microscopy (SEM). The experimental results indicate that the material’s gelation time can be controlled between 30s and 1600s, with an initial viscosity ranging from 5.9 to 9.8 mPa·s. Predictive models for these two indicators were established, and variance analysis revealed the influence ranking for gelation time: phosphoric acid dosage had the greatest effect, followed by EGDA content, with the Baume degree of sodium silicate having the least effect. The initial viscosity positively correlated with the Baume degree of sodium silicate and exhibited exponential growth over time. EGDA addition enhanced UCS by over 450%, reaching 1.2 MPa. During freeze-thaw cycles, strength degradation of the consolidated body was reduced by 10% to 30%. Microstructural tests showed that EGDA promotes silica gel formation and creates a network structure with unreacted sodium silicate, forming a dense consolidated body with moraine fine particles, thereby enhancing freeze-thaw resistance. These findings provide design references and theoretical support for moraine grouting in cold regions.

## 1. Introduction

In Shangri-La City, Yunnan Province, China, extensive moraine [1] deposits pose a significant risk of geological disasters such as landslides, debris flows, and uneven settlement [2,3,4] which seriously threaten the stability and safety of highways, railways, and slopes [5,6,7]. Grouting technology has been widely applied in fields such as civil engineering, construction, mining, and hydraulic engineering due to its ability to improve the physical and mechanical properties of geological bodies [8,9,10,11]. The selection of grouting materials and the performance of the consolidated body have become key areas of research focus [4,12,13].

Cement-based grouting materials are the most common choice due to their advantages of low cost, good mechanical properties, and wide availability of raw materials [14,15]. When the grouting medium has low permeability or contains a large amount of fine particles similar in size to cement particles, it becomes difficult for the cement to infiltrate and form a solidified structure [16,17], as seen in materials like clay, sandy soil, and moraine. Compared with cement-based grouting materials, chemical grouting materials have the advantages of low slurry viscosity, good pumpability, and adjustable gelation time [18], making them particularly well-suited for grouting reinforcement in moraine formations. Chemical grouting materials include epoxy resin, acrylate, and sodium silicate [19,20,21]. Considering factors such as performance, cost, environmental impact, and human safety, sodium silicate-based grouting materials are the optimal choice. However, the long gelation time and low cohesive strength of sodium silicate limit its application in engineering projects.

To optimize the gelation time and enhance the cohesive strength of sodium silicate grouting materials, many researchers have used additives such as phosphoric acid, boric acid, formamide, ethyl acetate, and glyoxal to improve their performance [22,23,24,25,26,27]. Wang et al. [22] developed an acidic grouting material using sodium silicate and phosphoric acid and investigated its setting time and viscosity variations. The findings revealed that the higher the Baume degree of sodium silicate, the shorter the setting time. Additionally, the optimal mix ratio for consolidating sand was recommended. Mollamahmutoğlu et al. [23] noted that with increasing amounts of sodium silicate and boric acid, the setting time decreases, viscosity rises, and their synergistic effect is enhanced. However, excessive boric acid further increases permeability. Avci et al. [24] investigated the impact of sodium silicate-formamide on the shear strength parameters of consolidated sand. The syneresis of sodium silicate-formamide grout gels increased with higher sodium silicate content, but only up to a certain point. Cui et al. [25] developed a high-performance material (DS grout) using sodium silicate and lipids. Their research showed that the setting time initially increased and then decreased as the amount of lipid curing agent was adjusted. By introducing a catalyst, the setting time could be effectively controlled. The material was successfully injected into sand layers and demonstrated superior consolidation performance compared to phosphoric acid-sodium silicate materials. Mollamahmutoğlu et al. [26] recommended two sodium silicate-glyoxal (SG) grout materials. The research indicated that these materials are naturally organic and environmentally friendly, offering excellent stabilization effects for cementing permeable silt and fine sand. Yu [27] introduced a new grouting material, Al_2_O_3_-WG/PU. Through response surface methodology, it was determined that Al_2_O_3_ reduces the reaction temperature by 12.7% and improves mechanical properties by 27.3%, providing a safer and more efficient solution for coal mine grouting.

Existing literature on sodium silicate grouting materials covers gelation time, viscosity control, and the effects of various additives; however, there is limited research on the performance of sodium silicate grout in low-temperature freeze-thaw environments, particularly on freeze-thaw durability. In the Qinghai-Tibet Plateau, western Sichuan Plateau, and Shangri-La region of China, the high altitudes, cold climates, and significant temperature fluctuations during winter lead to frequent freeze-thaw cycles [28,29,30]. These intense cycles cause a reduction in the strength of rocks and soils, structural damage, and the formation of cracks [31,32,33], thus requiring enhanced performance from grouting materials. To address this, our study introduces additives such as ethylene glycol diacetate (EGDA) into sodium silicate grout and examines their effects on gelation time and viscosity. Additionally, freeze-thaw cycle experiments assess the freeze-thaw resistance of the consolidated material. This research fills a gap in freeze-thaw durability studies on sodium silicate grouting materials in cold regions and provides essential references for material selection and design in practical engineering.

## 2. Materials and Methods

### 2.1. Raw Materials

#### 2.1.1. Grouting Materials

The grouting materials primarily include sodium silicate, curing agents, and catalysts, with specific material parameters as follows:

Sodium silicate: The sodium silicate was acquired from Kunming Yongfeng Chemical Co. in Kunming, Yunnan Province, China, with a Baume degree of 35 °Bé, a modulus of 3.26 and a density of 1.383 g/cm^3^, according to the Chinese standard (GB/T 4209-2008) [34].

Curing agent: The curing agent used in the tests was ethylene glycol diacetate (EGDA) provided by Anda Chemicals Co., Ltd. in Guiyang, Guizhou Province, China. It had a density of 1.106 g/cm^3^ and an ester content of 99% by reference to the Chinese standard (GB/T 4472-2011) [35].

Catalyst: The catalyst used in the tests was phosphoric acid provided by Changzhou Chuanlin Chemical Co., Ltd. in Changzhou, Jiangsu Province, China. With a phosphoric acid concentration of 85.2%, it meets the technical requirements of GB/T 2091-2008 [36].

#### 2.1.2. Moraine

**Figure 1:**
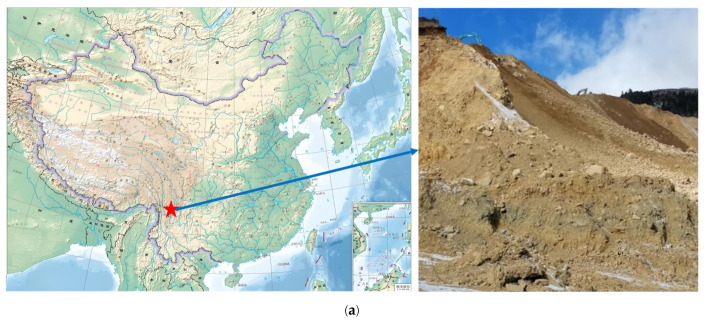

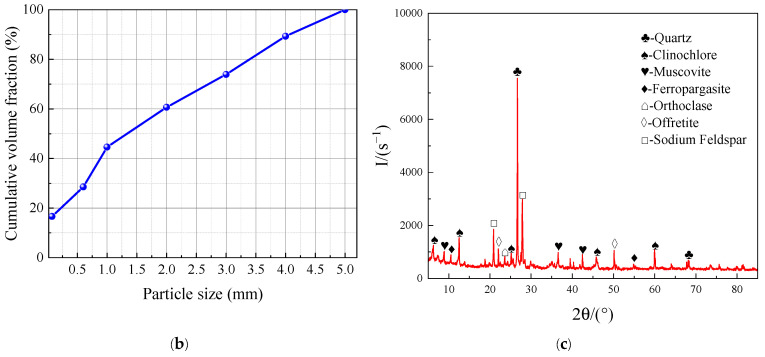
The particle characteristics of moraine. (**a**) The project site. (**b**) Particle size distribution. (**c**) XRD pattern of moraine.

The moraine used in this experiment was sourced from slope engineering at the Pulang Copper Mine in Shangri-La, Yunnan Province, as shown in Figure 1a. Shangri-La has a tundra climate (ET) according to the Köppen-Geiger classification, characterized by a low annual average temperature. Summers are mild, winters are cold, and temperatures vary significantly throughout the year, with marked differences between day and night. This study focused on the fine-grained fraction of the moraine, consisting of particles smaller than 5 mm. The fine-grained portion was sieved, and the particle gradation characteristics are shown in Figure 1b. The fine-grained portion accounts for approximately 40% of the total glacial sediment. As can be seen from the XRD pattern in Figure 1c, the main phases are quartz and sodium feldspar.

### 2.2. Experimental Design

#### 2.2.1. Slurry Mixing Procedure

The slurry consisted of equal volumes of solution A and solution B. Solution A was sodium silicate with varying degrees of Baume, diluted with water to obtain the desired concentration [37], as referenced in Equation (1). Solution B was a mixture of EGDA, phosphoric acid, and water, combined in ratios according to the experimental protocol. Solution B was poured into Solution A and stirred thoroughly until fully blended.

(1) $$M_{s}/M_{w}=\frac{B\left(145-A\right)}{145\left(A-B\right)}$$

where $M_{s}$ is the initial weight of the sodium silicate, $M_{W}$ is the weight of water added for dilution, *A* is the initial °Bé of the sodium silicate, and *B* is the target °Bé.

#### 2.2.2. Slurry Mix Design

The response surface methodology (RSM) experimental design was implemented using the Box-Behnken Design (BBD) in the Design-Expert software (version 13). As shown in Table 1, three experimental variables at three levels were selected. Gelation time and initial viscosity were chosen as the response variables.

#### 2.2.3. Sample Preparation

The samples used for the uniaxial compressive strength test were prepared by a mixing method where the moraine and slurry were thoroughly mixed into a uniform paste at a weight ratio of 6:1. This paste was then poured into cylindrical molds (50 mm in diameter × 100 mm in height) [38], and the molds were gently vibrated to eliminate any trapped air. The sample preparation design is presented in Table 2. The addition level of phosphoric acid was set at 3% to minimize its impact on strength enhancement.

**Figure 2:**
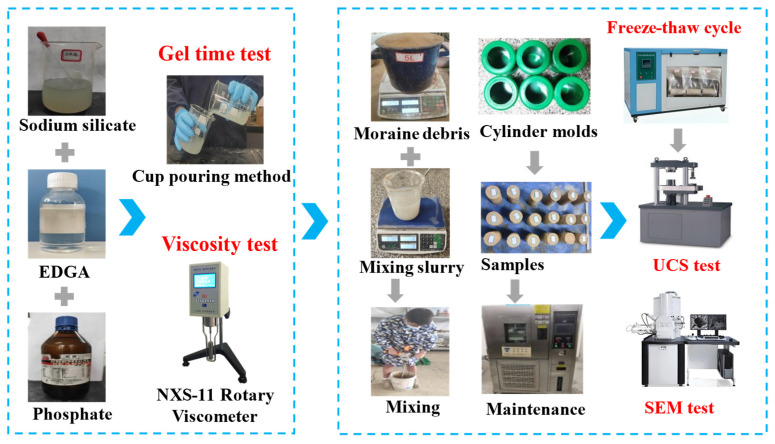
Experimental flowchart.

To investigate the effect of EGDA on enhancing the freeze-thaw resistance of consolidated bodies, various EGDA dosages and freeze-thaw cycle counts were selected as experimental variables. EGDA dosages were set at 0%, 5%, 10%, 15%, 20%, and 25%, while the number of freeze-thaw cycles varied between 0, 10, 30, and 50. After curing for 28 days, the freeze-thaw cycle tests were performed, followed by uniaxial compressive strength tests on the samples, with the experimental procedure illustrated in Figure 2.

### 2.3. Test Methods

#### 2.3.1. Gel Time Test

Gel time is a crucial indicator of grouting material performance. Following the JC/T 2536-2019 standard [39], the gel time was measured using the cup inversion method. Solution A and Solution B were placed in separate beakers at a specified volume ratio. Solution B was then poured into Solution A and thoroughly mixed, with timing initiated immediately. The mixture was continuously poured back and forth between the two beakers until it no longer flowed, at which point the recorded time was designated as the gel time of the slurry.

#### 2.3.2. Viscosity Tests

The time-dependent viscosity of the slurry is the basis for calculating the grouting diffusion range. In accordance with GB/T 22235-2008 [40], the slurry viscosity was tested using an NXS-11 rotational viscometer. The initial viscosity of the slurry was measured immediately after thorough mixing, and subsequent measurements were taken every 60 s until the viscosity exceeded 60 mPa·s.

#### 2.3.3. Freeze-Thaw Cycle Tests

**Figure 3:**
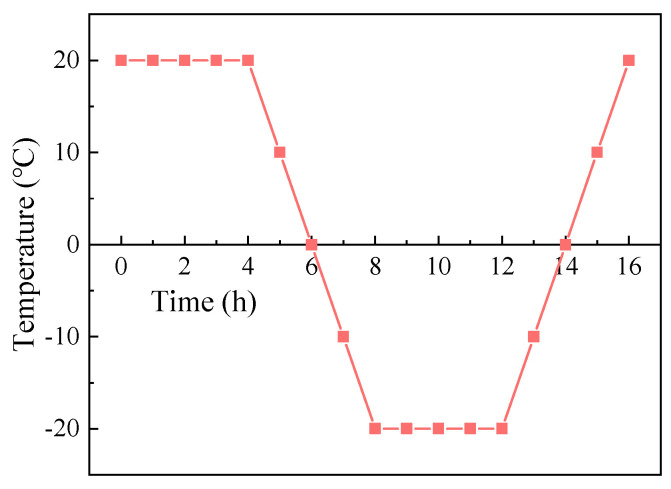
Freeze-thaw cycle design.

In accordance with GB/T 22235-2008, the samples cured for 28 days were placed in a freeze-thaw cycling machine. The freezing temperature was set at −20 °C, and the thawing temperature at 20 °C, with a temperature hold time of 4 h. The temperature transition time was also set to 4 h, making the duration of a single cycle 16 h. The design of a single freeze-thaw cycle is illustrated in Figure 3.

#### 2.3.4. UCS Tests

The UCS test was conducted using a TSMT300 (Shenzhen, China) testing machine, with a loading speed of 2 mm/min. To ensure the accuracy of the test results, three samples were tested in each group and the average value was taken as the final result, following the Chinese National Standard GB/T 17671-2021 [41].

#### 2.3.5. Microstructure Tests

The microstructure of the moraine consolidated body directly affects its macroscopic mechanical properties. XRD (D8-02, Bruker AXS, Karlsruhe, Germany), FTIR (Nexus 670, NICOLET, Madison, WI, USA) and SEM (Quanta 250, FEI, Hillsboro, OR, USA) were used for microscopic analysis to examine the main substances and microstructures in the consolidated body. XRD analysis was conducted within a 2θ range of 5° to 90°, with a step size of 0.02°.

## 3. Results and Discussion

### 3.1. Prediction Model

The test results for gel time and initial viscosity are presented in Table 3, where *Y* represents the measured values and Y* represents the predicted values. *Y*_1_ represents the gel time and *Y*_2_ represents the initial viscosity. The 17 sets of data were fitted using Design-Expert software to establish response models for gel time and initial viscosity, respectively. Through evaluating the accuracy of the models, we selected a multivariate nonlinear model and a linear model for representation, as illustrated in Equations (2) and (3), respectively.

Gel time (Quadratic):

(2) $$\begin{array}{l} Y_{1}=1400.0375+111.25X_{1}+50.46X_{2}-1189.75X_{3}+1.72X_{1}X_{2}+3.525X_{1}X_{3}\\ \;\;-0.725X_{2}X_{3}-3.504X_{1}^{2}-3.264X_{2}^{2}+136.0375X_{3}^{2}\\ \left(R^{2}=0.989\right) \end{array}$$

Initial viscosity (Linear):

(3) $$\begin{array}{l} Y_{2}=-0.428676+0.3225X_{1}+0.0225X_{2}-0.025X_{3}\\ \left(R^{2}=0.9357\right) \end{array}$$

The regression coefficients (*R*^2^) of the prediction models are 0.989 and 0.9357, respectively, indicating a high degree of model fit.

The reliability of the above regression equations was verified using analysis of variance (ANOVA). The results of the analysis are shown in Table 4.

The *F*-value is a parameter used to test the significance of the regression models. The *F*-values for the regression models are 69.76 and 63.02, respectively, indicating that the regression equations are significant. The *p*-value represents the probability of rejecting the null hypothesis, and both *p*-values are less than 0.01, indicating that the models have a high level of reliability. Therefore, the predicted values from the models show a strong correlation with the experimental values.

**Figure 4:**
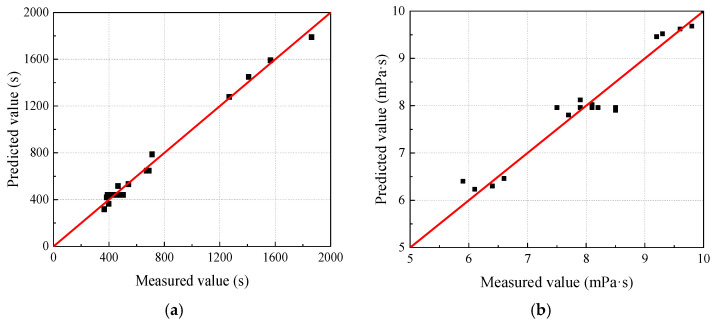
Comparison between measured and predicted values. (**a**) Gel time. (**b**) Initial viscosity.

The comparison between the measured and predicted values of gel time and initial viscosity is illustrated in Figure 4. All data points are located near the line y = x, further demonstrating the validity of the models.

### 3.2. Gel Time

#### 3.2.1. The Effect of Single Factors on Gel Time

**Figure 5:**
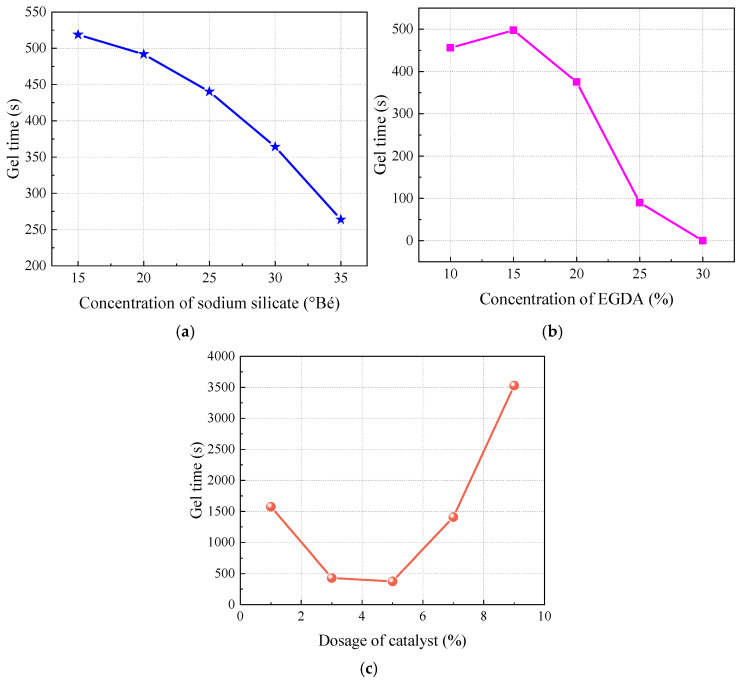
Influence of a single factor on gel time. (**a**) Concentration of sodium silicate. (**b**) Concentration of EGDA. (**c**) Dosage of catalyst.

As shown in Table 3, all factors have a significant impact on gel time. To analyze the effect of one factor on gel time, the other two factors can be fixed at a coded level of 0. Based on the regression model, the influence of phosphoric acid dosage and sodium silicate Baume degree on gel time was analyzed, as illustrated in Figure 5.

(1) The effect of Baume degree on gel time.

As depicted in Figure 5a, with the increase in concentration of sodium silicate, the gel time gradually decreases, and as the Baume degree increases, the reduction in gel time becomes more pronounced. This is because the increase of concentration of sodium silicate in the solution means more silica can be produced during the reaction, forming a gel network more quickly, which significantly shortens the gel time [42].

(2) The effect of concentration of EGDA on gel time.

As shown in Figure 5b, with the increase in EGDA dosage, the gel time gradually decreases, indicating that the addition of EGDA accelerates the gelation process. EGDA acts as a crosslinking agent capable of bridging multiple silicate molecules to form a crosslinked network structure. The addition of EGDA increases the crosslinking density of sodium silicate, thereby speeding up the formation of the gel network and making the gelation reaction occur more rapidly.

(3) The effect of dosage of catalyst on gel time.

As shown in Figure 5c, with the increase in phosphoric acid dosage, the gel time first decreases and then increases. The shortest gel time of 375 s occurs when the phosphoric acid dosage is 5%. This indicates that there is a specific range of phosphoric acid addition that results in the shortest gel time; further increasing the amount of phosphoric acid leads to a prolonged gel time. At an appropriate level of phosphoric acid addition, the acidic environment can promote the formation of silicate, thereby accelerating the gelation reaction. However, excessive phosphoric acid can make the system overly acidic, which affects the stability of colloidal particles, causing them to disperse and slowing down the gelation reaction. Additionally, an excess of phosphoric acid may combine with sodium ions in the sodium silicate, reducing the concentration of available sodium ions and inhibiting the formation of the gel network, thus extending the gel time.

#### 3.2.2. The Influence of the Interaction of Various Factors on Gel Time

**Figure 6:**
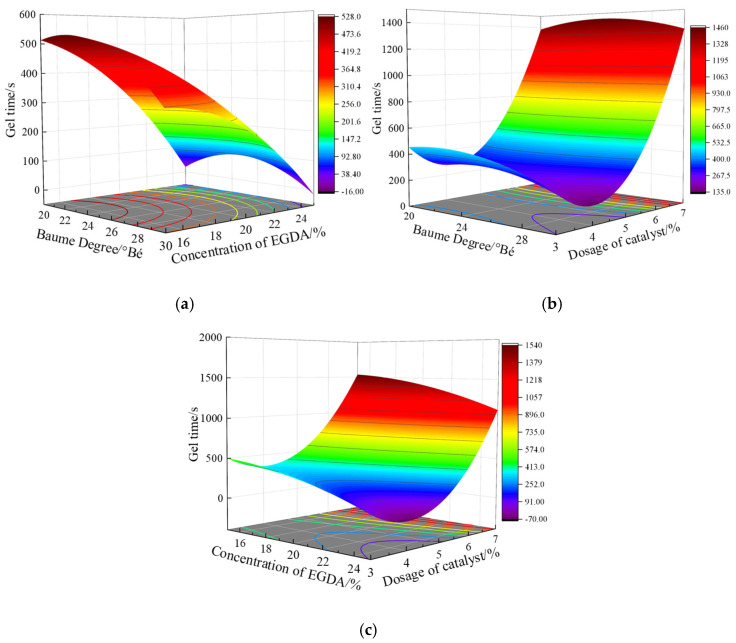
Interaction analysis between the factors. (**a**) *x*_1_*x*_2_ (**b**) *x*_1_*x*_3_ (**c**) *x*_2_*x*_3_.

As shown in Table 3, gel time is influenced not only by individual factors but also by the interactions between these factors. The significance of the interactions on gel time, in descending order, is *x*_1_*x*_2_, *x*_1_*x*_3_, and *x*_2_*x*_3_. Figure 6 illustrates the impact of these interactions on gel time.

By observing the surface shape in Figure 6a, it can be seen that gel time does not change linearly with the two factors; rather, it is influenced by a nonlinear combination of these factors. When the Baume degree is fixed, gel time significantly decreases with the increase in EGDA, although the rate of decrease gradually diminishes as the Baume degree rises. When the EGDA dosage is held constant, gel time first increases and then decreases with the increase in Baume degree. However, as the EGDA dosage increases, the peak of the curve shifts from a Baume degree of 22.25 °Bé to 24.19 °Bé. The difference at both ends of the curve decreases from 94.38 s to 0 s, indicating that the curve becomes increasingly symmetrical.

As shown in Figure 6b,c, during the interactions of *x*_1_*x*_3_ and *x*_2_*x*_3_, the phosphoric acid dosage (*x*_3_) plays a significant role, similar to the influence observed with phosphoric acid as a single factor.

#### 3.2.3. The Gelation Mechanism

Based on the magnitude of the *F*-values in the analysis of variance, it is evident that phosphoric acid has the most significant effect on gel time. Therefore, the variation in gel time can be explained by the gelation mechanism of sodium silicate in an acidic environment.

Phosphoric acid can provide $H^{+}$ for the reaction, which reacts with the $H_{2}{\mathrm{SiO}}_{4}^{2-}$ and $H_{2}{\mathrm{SiO}}_{4}^{3-}$ ions in the sodium silicate [43]. The reaction process is as follows:

(4) $$H_{2}{\mathrm{SiO}}_{4}^{2-}+H^{+}\to H_{3}{\mathrm{SiO}}_{4}^{-}$$

(5) $$H_{3}{\mathrm{SiO}}_{4}^{-}+H^{+}\to H_{4}{\mathrm{SiO}}_{4}$$

(6) $$H_{4}{\mathrm{SiO}}_{4}+H^{+}\to H_{5}{\mathrm{SiO}}_{4}^{+}$$

As the concentration of $H^{+}$ increases, the solution becomes acidic, causing the coordination number of silicate molecules to rise to 6. The silicate molecules then undergo a hydroxyl association reaction with monovalent silicate ions to form disilicate. The specific chemical reaction is as follows:

(7) $$H_{4}{\mathrm{SiO}}_{4}+H_{5}{\mathrm{SiO}}_{4}^{+}+2H_{2}O\to H_{13}{\mathrm{Si}}_{2}O_{10}^{+}$$

Disilicate subsequently polymerizes to form silica sol, and the particles of silica sol gradually increase in size during the gelation process. The particles agglomerate to form a gel network structure, with the formation of Si-O-Si bonds through this agglomeration, ultimately resulting in silica gel with a certain degree of strength.

At the same time, phosphoric acid can effectively protonate the ester groups, significantly accelerating their hydrolysis reaction to produce ethylene glycol and acetic acid. As a weak acid, acetic acid can release H⁺ ions in the reaction system, facilitating the hydrolysis of sodium silicate and the polymerization of silicate, which speeds up the overall curing process. The reaction is as follows:

(8) $$C_{6}H_{10}O_{4}+2H_{2}O\overset{H_{3}{\mathrm{PO}}_{4}}{\to }C_{2}H_{4}{\left(\mathrm{OH}\right)}_{2}+2{\mathrm{CH}}_{3}\mathrm{COOH}$$

When the concentration of ions in the solution reaches the optimal reaction value, the gel time is shortest. Conversely, an insufficient or excessive amount of $H^{+}$ will extend the reaction time.

### 3.3. Viscosity

#### 3.3.1. Initial Viscosity

The regression equation model and the analysis of variance results in Table 3 indicate that the initial viscosity of the slurry is linearly related to the factors, with the Baume degree having the most significant impact. In the early stages of slurry mixing, before the gelation reaction occurs, the initial viscosity primarily reflects the viscosity of the diluted sodium silicate.

#### 3.3.2. Time-Dependent Viscosity

**Figure 7:**
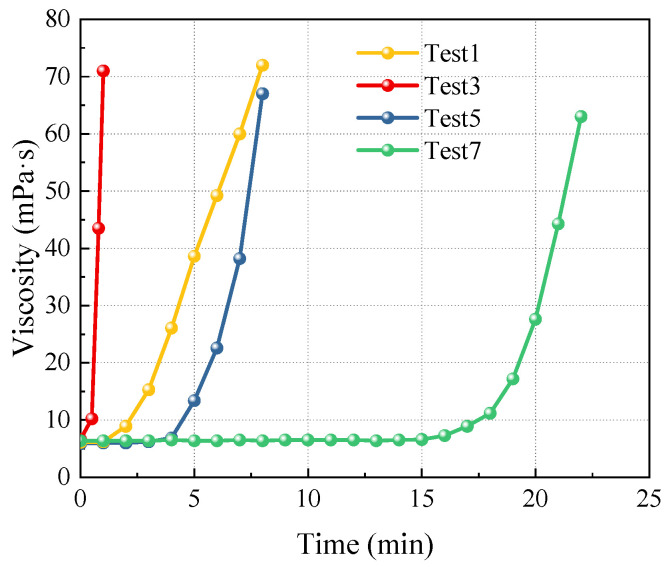
Time-varying curves of slurry viscosity.

The time-dependent viscosity of the slurry is a crucial indicator influencing grouting diffusion. A comparative analysis was conducted using the experiment at a Baume degree of 20°Bé, as illustrated in Figure 7.

Figure 7 shows that the time-dependent viscosity curves of the slurry exhibit an exponential growth pattern. Prior to gelation, the viscosity remains relatively low; however, it increases rapidly after a specific reaction time. Test 3 exhibited the shortest gel time, with the slurry viscosity increasing rapidly over time. Although Tests 1 and 5 had similar gel times, their viscosity growth patterns differed, as the viscosity growth rate in Test 1 was lower than in Test 5. This difference is due to the optimal phosphoric acid dosage in Test 1, which promotes an earlier onset of the gelation reaction. In contrast, the higher EGDA content in Test 5 further accelerates the gelation process, resulting in a more pronounced increase in viscosity. Test 7 maintained its initial viscosity for an extended duration because the excess phosphoric acid inhibited the gelation reaction.

### 3.4. Freeze-Thaw Resistance

#### 3.4.1. Mechanical Strength

**Figure 8:**
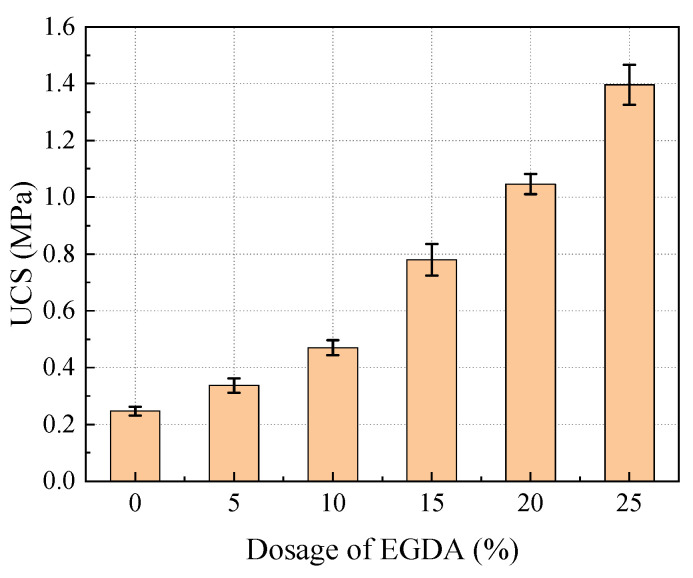
Effect of EGDA dosage on UCS before freeze-thaw cycle.

Figure 8 illustrates the UCS of moraine consolidated bodies with varying EGDA dosages before undergoing freeze-thaw cycling. The UCS exhibits a positive correlation with the amount of EGDA added. Notably, the UCS of the sample without any added EGDA was only 0.26 MPa., serving as the control group. As the EGDA dosage increased from 5% to 25%, the growth rates of UCS were recorded at 36%, 92%, 208%, 320%, and 456%, respectively. This demonstrates that the addition of EGDA significantly enhances the mechanical strength of the samples.

#### 3.4.2. Strength Attenuation

**Figure 9:**
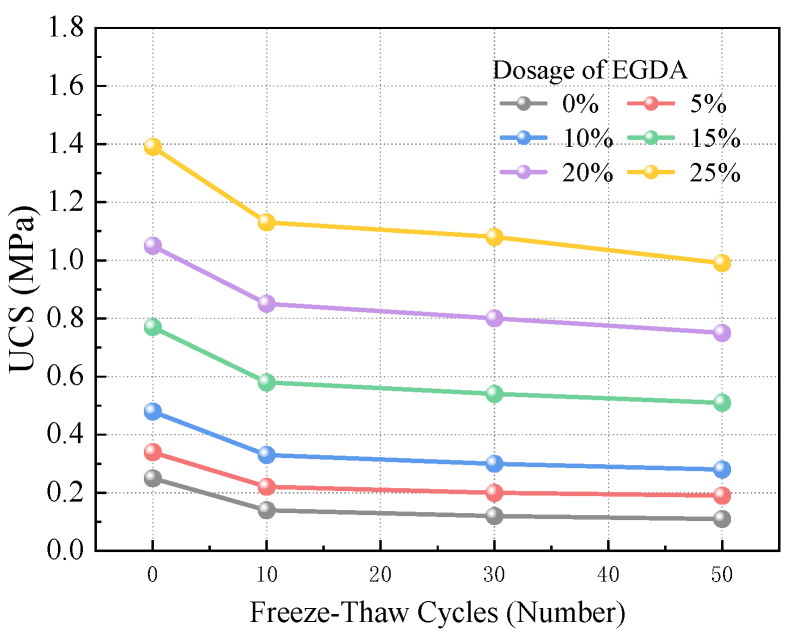
The effect of freeze-thaw cycles on UCS.

Figure 9 depicts the changes in UCS of the consolidated bodies after freeze-thaw cycling. The UCS initially declines rapidly and then decreases more gradually. After 10 freeze-thaw cycles, the UCS reduction percentages for EGDA dosages ranging from 0% to 25% were 44.00%, 35.29%, 31.25%, 24.68%, 19.05%, and 18.71%, respectively. From 10 to 50 cycles, the reduction percentages were 12%, 8.82%, 10.42%, 9.09%, 9.52%, and 10.07%. In the early stages of freeze-thaw cycling (≤10 cycles), the UCS declines sharply due to significant internal structural instability, leading to a rapid loss of load-bearing capacity. In contrast, in the later stages (>10 cycles), the UCS decreases more slowly as the remaining bonded structures stabilize after the failure of weaker components. The impact of freeze-thaw cycling on these stable structures is limited, allowing for better maintenance of load-bearing capacity. This suggests that after undergoing extensive freeze-thaw cycling, the internal damage of the consolidated body reaches a threshold, causing the UCS to stabilize at a certain value.

#### 3.4.3. Performance Improvement

**Figure 10:**
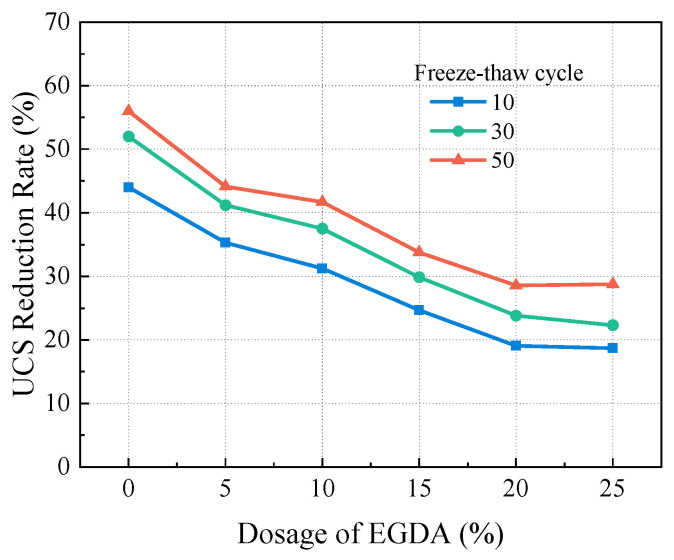
The effect of EGDA dosage on UCS after freeze-thaw cycles.

As shown in Figure 10, the strength attenuation rate decreases continuously with the increase in EGDA content. In the samples subjected to 30 freeze-thaw cycles, as the EGDA content increased from 5% to 25%, the UCS reduction rates were 41.18%, 37.50%, 29.87%, 23.81%, and 22.30%, respectively. In contrast, the sample without EGDA shows a reduction of 52%. Adding EGDA can improve the UCS by 10% at least. Clearly, incorporating EGDA effectively reduces the number of weak structures within the consolidated body, thereby improving its resistance to freeze-thaw cycles.

### 3.5. Solidification Mechanism

#### 3.5.1. XRD Analysis

**Figure 11:**
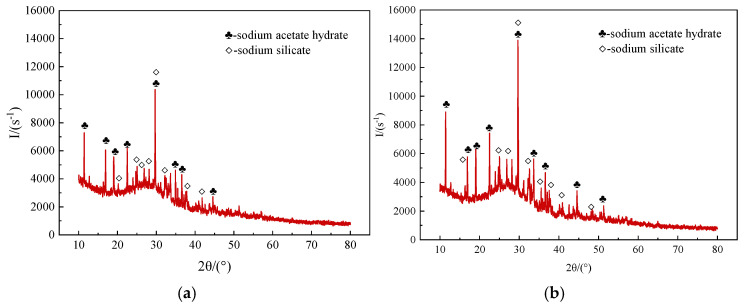
The XRD patterns of the consolidated bodies. (**a**) EGDA dosage of 5% (**b**) EGDA dosage of 15%.

To reveal the curing products and study the hardening mechanism of ester-modified sodium silicate grout materials, samples with 5% and 15% EGDA were selected for XRD testing. The XRD patterns are presented in Figure 11. Both samples exhibit two crystalline phases: sodium acetate hydrate and sodium silicate. Additionally, a diffuse peak of amorphous SiO_2_ appears in the 2θ range of 20° to 40°, indicating the presence of silica gel. The formation of sodium acetate hydrate is associated with the presence of acetic acid or an acidic environment during the reaction. Sodium silicate may indicate that some of the sodium silicate did not fully react, contributing to a complex network structure alongside the lipid. The formation of amorphous silica (silica gel) results from the polymerization reaction. The interactions among these components collectively influence the performance and application characteristics of the final gelation product.

#### 3.5.2. FTIR Analysis

**Figure 12:**
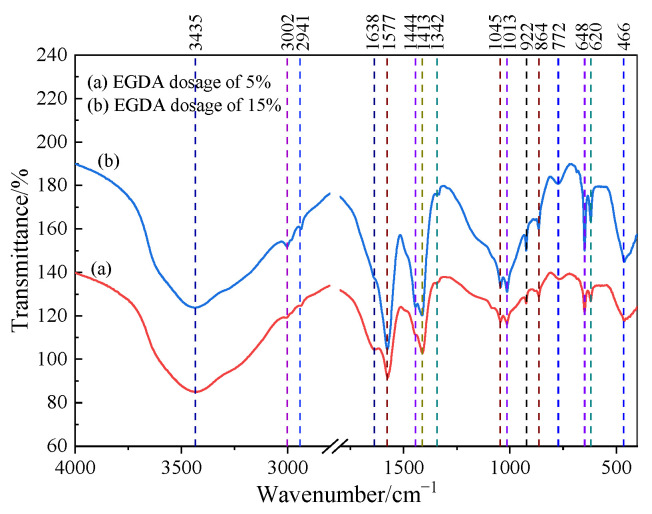
The FTIR spectrum.

FTIR testing provides a way to analyze both the chemical composition and structure. Figure 12 shows the FTIR spectra for two samples with the following key findings:

Water: A strong absorption at 3435 cm^−1^ signals O-H stretching from water bound in silica gel, while a weaker band at 1638 cm^−1^ is linked to O-H bending in free water.

Sodium acetate: Weak bands at 3002 cm^−1^ and 2941 cm^−1^ indicate C-H stretching in sodium acetate’s CH_3_ groups. Medium-strong bands at 1045 cm-1 and 1013 cm^−1^ are due to C-H rocking, with additional bands at 1577 cm^−1^, 1444 cm^−1^, and 1413 cm^−1^ showing C=O stretching in acetate ions.

Sodium silicate: The absorption bands at 1045 cm^−1^ and 1013 cm^−1^ are attributed to Si-O-Si stretching, while the band at 466 cm^−1^ is due to Si-O bending.

Amorphous Silica: A broad band at 772 cm^−1^ is associated with Si-O bond vibrations, indicating the presence of amorphous silica.

The analysis results confirm the presence of sodium acetate, sodium silicate, and amorphous SiO_2_ (silica gel) in the sample. These findings are consistent with the results obtained from the XRD analysis.

#### 3.5.3. SEM Analysis

**Figure 13:**
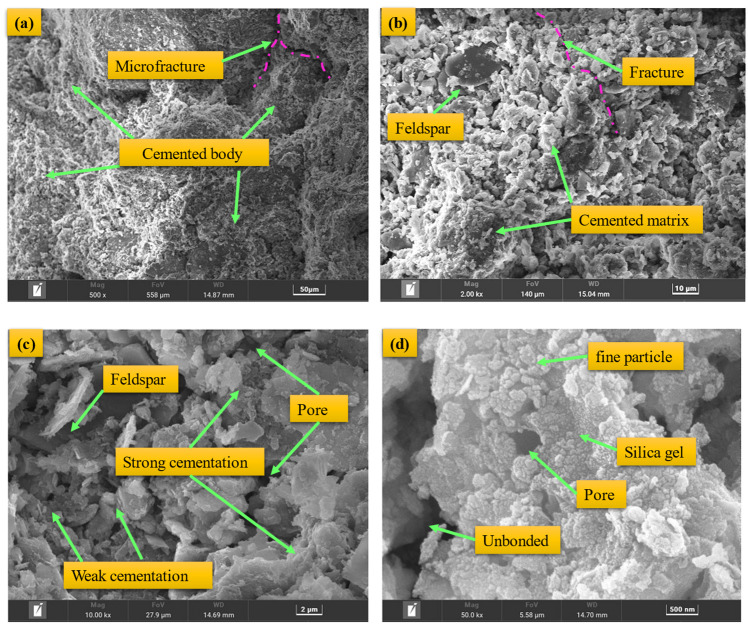
The SEM images of the moraine consolidation body after curing for 28 d: (**a**) 50 μm, (**b**) 10 μm, (**c**) 2 μm, (**d**) 500 nm.

Figure 13 shows the microstructure of the consolidated body with 15% EGDA addition after 28 days of curing and 30 freeze-thaw cycles. As seen in Figure 13a, the microstructure is relatively dense, with fine particles and grouting material forming a cemented matrix that fills the voids between large particles and bonds them together. Only a few microcracks are present in the consolidated body, which were caused by the freeze-thaw process.

From Figure 13b, it can be observed that the surface of the rock particles in the moraine is relatively smooth, while the surface of the clay minerals is coated with chemical consolidation materials. The cemented bodies are bonded to each other, forming a consolidated structure. Microcracks are distributed between the weakly cemented bodies. As shown in Figure 13c, there are structures with varying cementation strength between cemented bodies of different sizes, and some uncemented voids are also present. The distribution and quantity of these structures are the key factors determining the mechanical properties of the consolidated body. From Figure 13d, it can be further observed that the surfaces of the fine particles are covered and encapsulated by amorphous gel products. Uncemented areas show obvious voids or cracks.

The analysis of the consolidated body’s microstructure indicates that the strength and integrity of the cementation between fine particle agglomerates are key to improving the mechanical properties and freeze-thaw resistance of the consolidated body. This material exhibits outstanding mechanical strength and freeze-thaw resistance, making it ideal for infrastructure projects like tunnels, underground structures, and roads. The research fills a knowledge gap in the field, revealing a consolidation mechanism that offers new insights for material optimization. It promotes sustainable civil engineering, reduces maintenance costs, and enhances structural durability. Future studies could explore combinations of different modifiers for more efficient and eco-friendly solutions.

## 4. Conclusions

This paper studies the setting time and viscosity change patterns of ester-modified sodium silicate grouting materials. The freeze-thaw resistance of the moraine consolidation body was evaluated based on UCS variations. Additionally, the consolidation mechanism was analyzed through XRD, FTIR, and SEM. The following conclusions can be drawn:

(1) Ester-Modified Sodium Silicate Grout Material has the advantages of controllable gel time and adjustable viscosity, and the established predictive model can accurately guide the material mix design.
(2) The impact of various factors on gel time is significant, with the most notable factor being the amount of phosphoric acid added. As the amount of phosphoric acid increases, the gel time initially decreases and then starts to increase. The initial viscosity was positively correlated with the Baume degree of sodium silicate, and the viscosity exhibited an exponential increase over time.
(3) The addition of EGDA can effectively enhance the mechanical strength and freeze-thaw resistance of the samples. The UCS shows a rapid decrease at first, followed by a slower decline as the number of freeze-thaw cycles increases. The inclusion of EGDA reduces the strength attenuation of the consolidated body, with a reduction rate of 10% to 30%.
(4) The network structure formed by silica gel and lipids encapsulates fine particles, creating a dense consolidated body, which is the main reason for the enhancement of mechanical strength and freeze-thaw resistance. Freeze-thaw cycles disrupt the weaker cementation within the consolidated body, leading to a decrease in mechanical strength. A reasonable amount of EGDA can be selected based on engineering requirements, and the optimal ratio can be recommended using the predictive model.

## Figures and Tables

**Table 1:** RSM-BBD experimental factors and levels.

Factors	Levels		
	−1	0	1
*X*_1_: Baume degree/°Bé	20	25	30
*X*_2_: EGDA/%	15	20	25
*X*_3_: Phosphoric acid/%	3	5	7

**Table 2:** Sample preparation design.

Moraine: Slurry	Slurry		
	Baume Degree/°Bé	Phosphoric Acid/%	EGDA Dosages/%
6:1	25	3	0, 10, 15, 20, 25

**Table 3:** Test design and results of response surface methodology.

Serial Number	Design Value			Measured Value		Predicted Value	
	*X*_1_	*X*_2_	*X*_3_	*Y*_1_	*Y*_2_	*Y*_1_*	*Y*_2_*
1	20	15	5	468	6.1	512.25	6.23
2	30	15	5	260	9.2	307.00	9.46
3	20	25	5	66	6.6	19.00	6.46
4	30	25	5	30	9.8	0	9.68
5	20	20	3	466	5.9	438.13	6.40
6	30	20	3	279	9.6	248.38	9.62
7	20	20	7	1314	6.4	1344.63	6.30
8	30	20	7	1268	9.3	1295.88	9.52
9	25	15	3	562	8.5	545.63	7.90
10	25	25	3	78	7.9	152.88	8.12
11	25	15	7	1612	7.7	1537.13	7.80
12	25	25	7	1099	8.1	1115.38	8.02
13	25	20	5	291	7.5	375.20	7.96
14	25	20	5	332	8.2	375.20	7.96
15	25	20	5	402	8.5	375.20	7.96
16	25	20	5	452	8.1	375.20	7.96
17	25	20	5	399	7.9	375.20	7.96

Note: When the predicted value for gel time is negative, it is treated as 0.

**Table 4:** Variance analysis of response surface regression model.

Source	Sum of Squares		Mean of Squares		*F*-Value		*p*-Value	
	*Y*_1_	*Y*_2_	*Y*_1_	*Y*_2_	*Y*_1_	*Y*_2_	*Y*_1_	*Y*_2_
Model	3.56 × 10^6^	20.92	3.95 × 10^5^	6.97	69.76	63.02	<0.001	<0.001
*x*_1_	28,441.12	20.80	28,441.12	20.80	5.02	187.96	0.06	<0.001
*x*_2_	3.32 × 10^5^	0.10	3.32 × 10^5^	0.1013	58.56	0.91	<0.001	0.36
*x*_3_	1.91 × 10^6^	0.02	1.91 × 10^6^	0.020	337.02	0.18	<0.001	0.68
*x*_1_ *x*_2_	7396.00		7396.00		1.31		0.29	
*x*_1_ *x*_3_	4970.25		4970.25		0.88		0.38	
*x*_2_ *x*_3_	210.25		210.25		0.04		0.85	
*x*_1_^2^	32,310.57		32,310.57		5.70		0.05	
*x*_2_^2^	28036.04		28,036.04		4.95		0.06	
*x*_3_^2^	1.25 × 10^6^		1.25 × 10^6^		220.10		<0.001	
Residual	396,651.55	1.44	5664.51	0.1107				
Lack of fit	23,512.75	0.89	7837.58	0.0985	1.94	0.71	0.27	0.69
Pure Error	16,138.80	0.550	4034.70	0.1380				
Cor Total	3.60 × 10^6^	22.36						

## Data Availability

The original contributions presented in the study are included in the article; further inquiries can be directed to the corresponding author.
